# The Evolution of Pediatric MAFLD Research (2014-2023): A Decade-Long Bibliometric Analysis of Emerging Trends

**DOI:** 10.2174/0118715303404437250611123553

**Published:** 2025-07-07

**Authors:** Tianyi Li, Xiaoying Zhang, Daojun Wang, Lixia Zhang, Qiong Wu, Wei Yan, Fengfeng Cui, Mengyao Huang, Peng Hua, Xiang Cui

**Affiliations:** 1 Department of Hepatology, Ankang Hospital of Traditional Chinese Medicine, Ankang, 725000, Shaanxi, China;; 2 Key Laboratory of Invigorating Spleen and Turbid Method for Metabolism-related Fatty Liver Disease of Shaanxi Provincial Administration of Traditional Chinese Medicine, Ankang, 725000, Shaanxi, China;; 3 First Clinical Medical College of Shaanxi University of Chinese Medicine, Xianyang, 712046, Shaanxi, China;; 4 Food Technology Division School of Industrial Technology Universiti Sains Malaysia 11800 Minden Penang, Malaysia;; 5 Ankang Nutrition Society, Ankang, 725000, Shaanxi, China

**Keywords:** MAFLD, children, bibliometric analysis, NAFLD, CVD, VOSviewer

## Abstract

**Background:**

Metabolic dysfunction-related fatty liver disease (MAFLD) has emerged as the predominant chronic liver disorder among children and adolescents. Like in adults, pediatric MAFLD encompasses a disease spectrum progressing from isolated steatosis to inflammatory changes, fibrotic development, and ultimately, cirrhosis. Despite increasing recognition of MAFLD as a major pediatric health issue, current literature lacks a systematic quantitative evaluation of research trends, leading to knowledge gaps in this field. To address this limitation, a comprehensive bibliometric analysis was performed to assess global research output on pediatric MAFLD by focusing specifically on the 2014-2023 period. This analysis avoids the confounding effects of the heterogeneity of earlier data while achieving sufficient temporal resolution to reveal emerging trends that might be obscured in long-term studies. This study synthesizes existing evidence, enhances understanding of this disciplinary field, and informs future research directions in pediatric MAFLD.

**Methods:**

Articles concerning children with MAFLD published from 2014-2023 were identified from the Science Citation Index-Expanded of the Web of Science Core Collection. CiteSpace software, VOSviewer, and the Online Analysis Platform of Literature Metrology were used to analyze the current publication trends and hotspots.

**Results:**

The analysis identified 1,609 English-language articles on pediatric MAFLD published from 2014 to 2023. The United States emerged as the most active participant in international collaborations. The University of California San Diego (UCSD) demonstrated the highest research output among the analyzed institutions. Additionally, UCSD exhibited the most extensive collaborative network, engaging in frequent and substantive research partnerships with a diverse range of academic and scientific organizations. Valerio Nobili was found to be the most prolific author, with 67 articles. Keyword burst analysis revealed that cardiovascular risk factors were the most intense research hotspot.

**Conclusion:**

Current research on pediatric MAFLD warrants greater attention, particularly regarding cardiovascular risk factors. This study provides valuable references for researchers, offering insights to guide future research directions and potential collaborations.

## 1 INTRODUCTION

Although pediatric non-alcoholic fatty liver disease (NAFLD) has been documented since the 1980s [1, 2], the field long suffered from inconsistent diagnostic criteria (*e.g.*, the upper limit of ALT varies greatly and is set too high [3], unclear natural disease courses, the lack of non-invasive diagnostic tools, and the lack of approved guidelines [4]). The NASPGHAN guideline working group has put forward suggestions for guiding the screening and clinical care of pediatric NAFLD [5], marking an important shift in pediatric NAFLD research and clinical practice. Metabolic dysfunction-related fatty liver disease, previously referred to as nonalcoholic fatty liver disease (NAFLD), represents a significant public health concern and is the most prevalent chronic liver condition globally. In 2020, an international expert consensus panel proposed a pivotal nosological revision for metabolic dysfunction-associated fatty liver disease (MAFLD) in adults. This redefinition shifts from the historically used term “NAFLD”—which carries a purely exclusionary connotation—to the more clinically precise and mechanistically grounded term “MAFLD”. This change underscores the condition’s intrinsic link to metabolic dysregulation, emphasizing its pathogenesis within the spectrum of metabolic syndrome rather than merely the absence of alcohol consumption [6]. This shift underscores the condition's metabolic origins and aligns it more closely with underlying metabolic dysfunction. This change has also spurred increased interest in elucidating the pathophysiology, risk factors, and potential interventions for pediatric MAFLD [7-9].

The global prevalence of MAFLD among children is estimated to range from 7% to 35%, contingent upon diagnostic methodologies and population characteristics. This condition exhibits a high prevalence in obese children, affecting an estimated 34.2% of this population [10]. Existing evidence suggests an association between childhood obesity and MAFLD, with obesity identified as a potential risk factor for MAFLD development in pediatric populations [11-15]. Early intervention through lifestyle modification and targeted pharmacological treatment is imperative to mitigate the long-term health risks associated with both conditions. Consequently, it has emerged as one of the primary causes of chronic liver disease in children and adolescents worldwide, largely attributable to rising obesity rates within these age groups [16]. MAFLD commonly presents as a component of a multisystem metabolic disorder, frequently coexisting with multiple comorbidities. These include type 2 diabetes mellitus (T2DM), dyslipidemia, hypertension, hypothyroidism, cholelithiasis, gastroesophageal reflux disease (GERD), obstructive sleep apnea (OSA), and depressive disorder. The presence of these concurrent conditions substantially elevates the disease burden and contributes to higher patient morbidity rates [17, 18]. Notably, the global prevalence of pediatric metabolic dysfunction is rising in parallel with obesity trends. According to World Health Organization (WHO) surveillance data, an estimated 39 million children under five years of age were classified as overweight or obese in 2020 [19], underscoring the growing public health challenge of obesity-related metabolic complications, including MAFLD. Furthermore, the prevalence of MAFLD among children and adolescents globally increased from an estimated 19.34 million cases in 1990 to approximately 29.49 million cases by 2017 [20]. Over the past two decades, instances of fatty liver disease among children and adolescents in the United States have more than doubled; this condition has now become one of the leading causes of chronic liver diseases across numerous countries [21]. Children and adolescents can present with a wide spectrum of liver disease severities, encompassing steatosis, steatohepatitis—either with or without fibrosis—and end-stage cirrhosis [22]. Reports documenting fatty liver disease in pediatric populations are derived primarily from retrospective case series [23]. Emerging epidemiological evidence demonstrates that elevated body mass index (BMI) during childhood constitutes a significant independent risk factor for progressive liver disease in later life. These studies have established a strong association between pediatric obesity and the subsequent development of severe hepatic complications, including cirrhosis and hepatocellular carcinoma (HCC), in adulthood. This association persists even after adjustment for potential confounding factors, suggesting a direct pathophysiological link between early-life metabolic dysfunction and long-term hepatic morbidity [24].

The prevalence of MAFLD and the related morbidity and mortality among children are anticipated to increase further in the coming years. Currently, this condition has significant social implications; however, it remains insufficiently recognized and inadequately assessed. Childhood MAFLD often manifests early, exhibiting a histological severity comparable to that observed in adults. Consequently, affected children may experience advanced liver disease as they transition into early adulthood, exacerbating comorbidities such as metabolic syndrome and cardiovascular diseases [25]. There is a complex interplay between fatty liver disease and other prevalent conditions—including cardiovascular diseases-which represent the leading cause of mortality among individuals afflicted by fatty liver disease. This relationship is mediated through cardiometabolic factors alongside various risk factors; moreover, extrahepatic complications increase as the severity of hepatic impairment increases [26]. The issue of MAFLD in children has become increasingly critical. Although promising therapeutic options are emerging on the horizon, patients continue to face substantial challenges owing to limited data regarding the natural history of this condition within this demographic. Addressing this escalating epidemic among children will undoubtedly yield positive outcomes for adult MAFLD management while alleviating its associated social and economic burdens [6].

The widespread global prevalence of child-related MAFLD has imposed a significant burden on healthcare systems and created substantial economic challenges, thereby necessitating an increased focus on in-depth research in this area. However, over the past decade, few bibliometric analyses summarizing this topic have been conducted. Current research predominantly focuses on adult MAFLD, with pediatric MAFLD studies remaining relatively limited and primarily employing broad temporal frameworks [27-30]. Such approaches may obscure critical period-specific research trends. By concentrating specifically on the 2014-2023 timeframe, our analysis circumvents interference from earlier data heterogeneity while revealing emerging patterns that might be masked in long-term studies. Bibliometric analysis serves as a powerful scientometric tool that enables both quantitative assessment and qualitative evaluation of research development within a defined temporal framework. This approach provides valuable insights into the intellectual structure of the MAFLD research domain, identifies emerging knowledge clusters, and delineates the evolution of research hotspots over time. The findings will offer researchers a systematic overview of the current knowledge landscape and facilitate the identification of future research directions in this rapidly evolving field. This study conducts a comprehensive bibliometric analysis of pediatric fatty liver disease research spanning the decade before and after the NAFLD-to-MAFLD nosological transition (2014-2023). Specifically, the aim is to: (1) examine the evolving research landscape across this terminology shift period through quantitative publication analysis, (2) quantify global research disparities and institutional collaboration structures using co-authorship analysis, and (3) identify emerging research trends through keyword evolution analysis. The findings will provide a systematic assessment of the field's development during this critical nosological transition period.

## 2 MATERIALS AND METHODS

### 2.1 Data Sources and Search Strategies

A comprehensive literature search was conducted within the Science Citation Index-Expanded (SCI-E) of the Web of Science Core Collection (WoSCC) database for the period of 2014-2023 on November 16, 2024. To minimize potential bias introduced by database updates, all search queries and data extraction were executed within a single day. Our systematic search strategy employed the Web of Science Core Collection (WoSCC) database with the following parameters: TS=(“NAFLD” or “nonalcoholic fatty liver disease” or “MAFLD” or “metabolic dysfunction-associated fatty liver disease” or “metabolic associated fatty liver disease” AND “children”) from January 1, 2014, to December 31, 2023, and the “Document Type” was limited to “Articles” only. To ensure the reliability of our bibliometric analysis, a rigorous three-stage screening process was implemented: (i) Initial title screening, (ii) Abstract review, and (iii) Full publication year verification.

Exclusion criteria were systematically applied as follows: (i) Publications unrelated to MAFLD pathophysiology, diagnosis, or management, (ii) Non-article document types (including editorials, letters, conference proceedings, and commentaries), (iii) Duplicate publications, and (iv) Non-English language articles. The literature screening was performed independently by two researchers, with any disagreements resolved through neutral arbitration by chief physicians to maintain process transparency and impartiality. Following this comprehensive screening process, a final cohort of 1,609 articles meeting all inclusion criteria was identified for subsequent bibliometric analysis.

### 2.2 Bibliometric Analysis

To systematically characterize the global research landscape of pediatric MAFLD studies, a multi-platform bibliometric approach was employed utilizing the following analytical tools and procedures: (i) Data Acquisition and Preprocessing. A comprehensive literature retrieval was conducted through the Web of Science (WoS) Core Collection (http://wcs.webofknowledge.com). Temporal publication trends were extracted using built-in WoS analytical functions, and all eligible records were exported in plain text (TXT) format for subsequent analysis. (ii) Analytical Platforms. All the data that met the requirements were converted from WOSCC and imported into CiteSpace analysis software 5.7.R5 (Drexel University, Philadelphia, PA, USA). This software, developed by Professor Chaomei Chen, is used for visual co-citation network analysis and burst detection [31]. VOSviewer 1.6.18 (Leiden University, Leiden, The Netherlands) is a software tool for constructing and visualizing bibliometric networks and is often used to summarize the most prolific countries/regions, institutions, journals, and authors (see www.vosviewer.com). In this study, VOSviewer was used to show the top 10 most cited journals and achievements of different countries/regions and institutions. This analytical approach performed a temporal trend analysis of publication outputs, generated co-authorship networks to examine collaboration patterns, implemented co-citation analysis to identify knowledge foundations, and executed keyword co-occurrence analysis to detect research hotspots. This integrated analytical framework enables comprehensive evaluation of the pediatric MAFLD research domain across multiple dimensions, including temporal evolution, institutional contributions, and structure.

CiteSpace was configured with the following analytical parameters: (i) Temporal segmentation: 2014-2023 (yearly slices), (ii) Term source selection: Full-text analysis, (iii) Ode types: Sequentially analyzed (author, institution, country, reference), (iv) Network pruning: Pathfinder algorithm, and (v) Visualization mode: Static cluster view with merged networks. In the knowledge map generated by CiteSpace, nodes represent discrete bibliometric entities (author, institution, country, reference), while links between nodes represent cooperative/co-occurrence or co-citation relationships. Using the bibliometrics online analysis platform, the top 10 productive countries by number of publications and the top 10 active journals by number of publications were extracted.

## 3 RESULTS

### 3.1 Quantity and Trends Analysis of Published Papers

As shown in Fig. (**1**), in the SCI-E of the WoSCC, the total number of papers published from 2014-2023 that met our inclusion criteria was 3,276. Following rigorous application of our predefined exclusion criteria, 1,667 publications that did not meet our specified article type requirements were systematically excluded, including review articles, meta-analyses, litigation papers, and correction articles. Through this standardized screening process, 1,609 relevant research articles were identified and extracted from the Web of Science Core Collection (WoSCC) that fully complied with our established inclusion criteria for subsequent bibliometric analysis. As shown in Fig. (**2**), there has been a slight increase in the number of publications over the last decade, indicating a gradual increase in interest in childhood MAFLD as a public health problem worthy of study.

### 3.2 Analysis of Intercountry/Region, Interinstitutional and Interauthor Cooperation

**Fig. (3):**
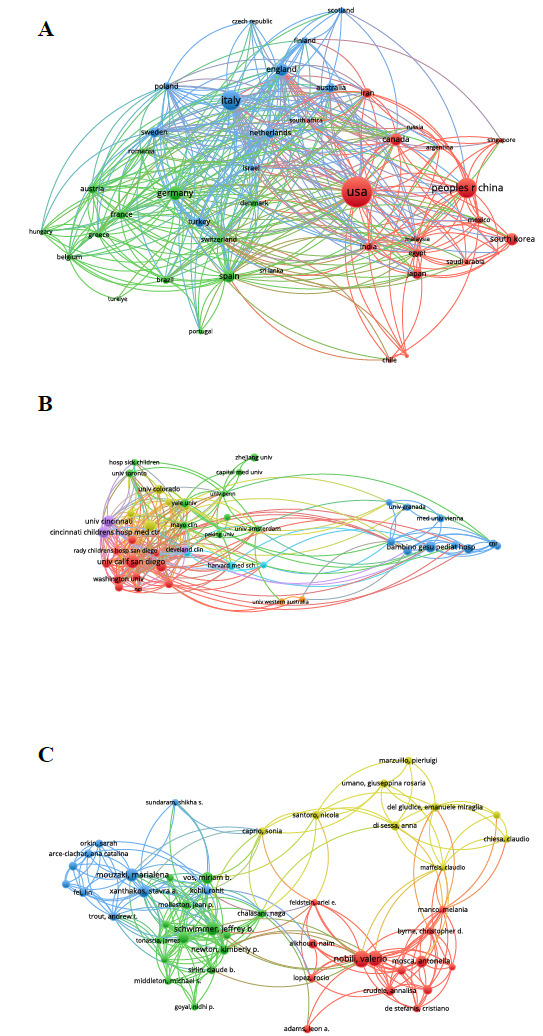
Visualization knowledge maps of authors, institutions, and countries/regions related to children's MAFLD from 2014-2023. (**A**) Cooperation map of countries/regions (total link strength = 1340). (**B**) Cooperation map of institutions (total link strength values = 986). (**C**) Cooperation map of the authors (total link strength values = 1415). Different colors indicate different clusters, and the node size indicates the number of publications. The thickness of the lines represents the link strength of the authors, countries/regions, and institutions.

The network visualization maps of the cooperation relationships among authors, organizations, and countries are shown in Fig. (**3**). The analysis encompassed publications originating from researchers across 80 distinct countries and regions. To ensure meaningful representation in our network analysis, a threshold of ≥ 5 publications per country was established. (Fig. **3A**) illustrates the collaborative network among the top 43 most productive countries/regions meeting this criterion. In the network visualization graph, nodes represent individual countries or regions, and their diameters are proportional to the output of publications. The connecting lines denote international collaborative relationships, and their thickness corresponds to the intensity of collaboration. The major findings of this analysis indicate that the United States is the most prolific contributor, having displayed both the highest volume of publications and maintaining the broadest collaborative network. Moreover, Fig. (**3B**) shows 46 institutions with 15 or more collaborations. It can be seen from the collaboration chart that Univ Calif San Diego has the largest number of published papers and the most frequent collaborations with other institutions. Furthermore, (Fig. **3C**) illustrates 48 authors whose number of collaborations is greater than or equal to 10. It can be seen from the collaboration graph that Valerio Nobili is the author with the most cooperation and communication.

To identify the leading contributors to the advancement of this research domain over the past decade, a bibliometric analysis of publication outputs was conducted across nations and regions. As presented in Table **1**, the analysis reveals the top ten most prolific countries/regions in terms of scholarly production. The results demonstrate that the United States maintains a dominant position, with Italy and China ranking second and third, respectively. Notably, statistical analysis indicates that the U.S. publication output significantly surpasses that of all other nations/regions.

### 3.3 Analysis of Coauthorship Networks and Core Author Distribution

**Fig. (4):**
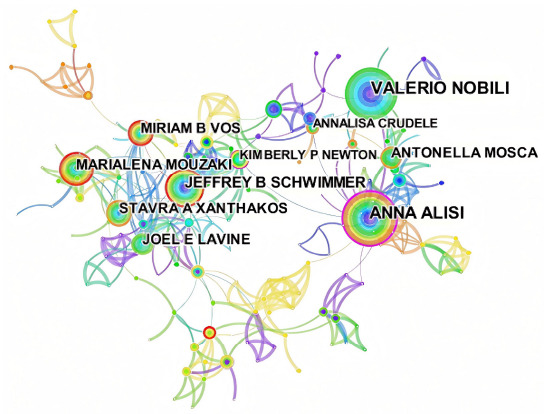
The visualization depicts the 10 most prolific authors in the field, with network nodes representing individual researchers and edges denoting collaborative relationships between them.

Over the past decade, 9,463 researchers have engaged in MAFLD research. Among these contributors, 48 leading authors (each with 10 or more publications) have played a pivotal role in advancing scholarly output. (Fig. **4**) illustrates the co-authorship network of the top 10 most prolific authors, where nodes represent individual researchers and connecting lines denote collaborative relationships. Prof. Valerio Nobili (Hepato-Gastroenterology Disease Unit, “Bambino Gesù” Children's Hospital IRCCS, Rome, Italy) emerged as the most published author, demonstrating the highest research productivity in this field (67), followed by Anna Alisi from the Research Unit of Molecular Genetics of Complex Phenotypes, “Bambino Gesù” Children's Hospital IRCCS, Rome, Italy (57). According to the cooperative network map of authors, the top 10 productive authors presented close collaborative relationships.

### 3.4 Analysis of Keywords

The co-occurrence of keywords, which can be classified into four clusters, is shown in Fig. (**5**), which presents the frontiers, trends, and hot topics in this field. The green cluster includes NAFLD and inflammation. The red cluster includes children, including those with obesity, those with insulin resistance, and those with metabolic syndrome. The blue cluster includes diagnosis, nonalcoholic fatty liver disease and steatohepatitis. The yellow cluster includes disease and nonalcoholic fatty liver. The top 10 keywords with the highest frequency were children, obesity, insulin resistance, metabolic syndrome, NAFLD, prevalence, adolescents, fatty liver disease, and hepatic steatosis.

### 3.5 Analysis of Journals

The original articles comprised publications disseminated across 493 scholarly journals. To evaluate the relative impact of these publication venues, a systematic bibliometric assessment was conducted using the bibliometric online analysis platform. The top 10 most-cited journals are shown in Table **2**, indicating that articles published in the Journal of Pediatric Gastroenterology and Nutrition were cited most frequently (436 times) during the past 10 years, followed by those in the Journal of Pediatrics (270), Hepatology (245), Gastroenterology (175), Plos One (155), Pediatric Obesity (148), the Journal of Hepatology (138), the World Journal of Gastroenterology (100), JAMA Pediatrics (81), and BMC Pediatrics (80). Five of these journals are from the United States, while the others are from the Netherlands, China, Denmark, Australia, and England. Articles published in Gastroenterology had the highest average number of citations per paper (11.67).

### 3.6 Analysis of Document Cocitation and Clustered Networks

Document co-citation analysis, a well-established bibliometric method, was employed to examine connections within the MAFLD research domain. This method evaluates relationships between publications by visualizing their co-occurrence of citations. This analysis identified references exhibiting strong citation bursts, indicative of particularly influential works. As depicted in Fig. (**6A**), 20 references are presented, demonstrating the most pronounced citation bursts. “Begin” denotes the initial year of citation detection, and “end” marks the termination point of the burst period. Notably, seven of these highly cited references continue to demonstrate sustained academic influence, suggesting their ongoing relevance to MAFLD research.

Fig. (**6B**) shows the coreference CiteSpace diagram for keyword clustering. Cocited cluster analysis revealed that the terms most relevant to MAFLD studies in children by stratified cluster labels included #0 cardiovascular risk factor, #1 secondary intestinal bacterial overgrowth, #2 liver disease, #3 insulin sensitivity, #4 fat fraction, #5 genetic determinant, #6 pediatric nonalcoholic fatty liver disease, #7 hepatocellular carcinoma, #8 UTR polymorphism, and #9 maternal high-fat diet. The analysis revealed an inverse relationship between cluster designation numbers and article volume, with lower-numbered clusters containing substantially more publications than higher-numbered clusters. The color gradient visualization demonstrates temporal citation patterns, where increasingly red hues indicate clusters receiving more frequent citations in recent years, suggesting these represent emerging or currently active research fronts in MAFLD investigation.

### 3.7 Analysis of Research Trends and Burst Detection with Keywords

**Fig. (7):**
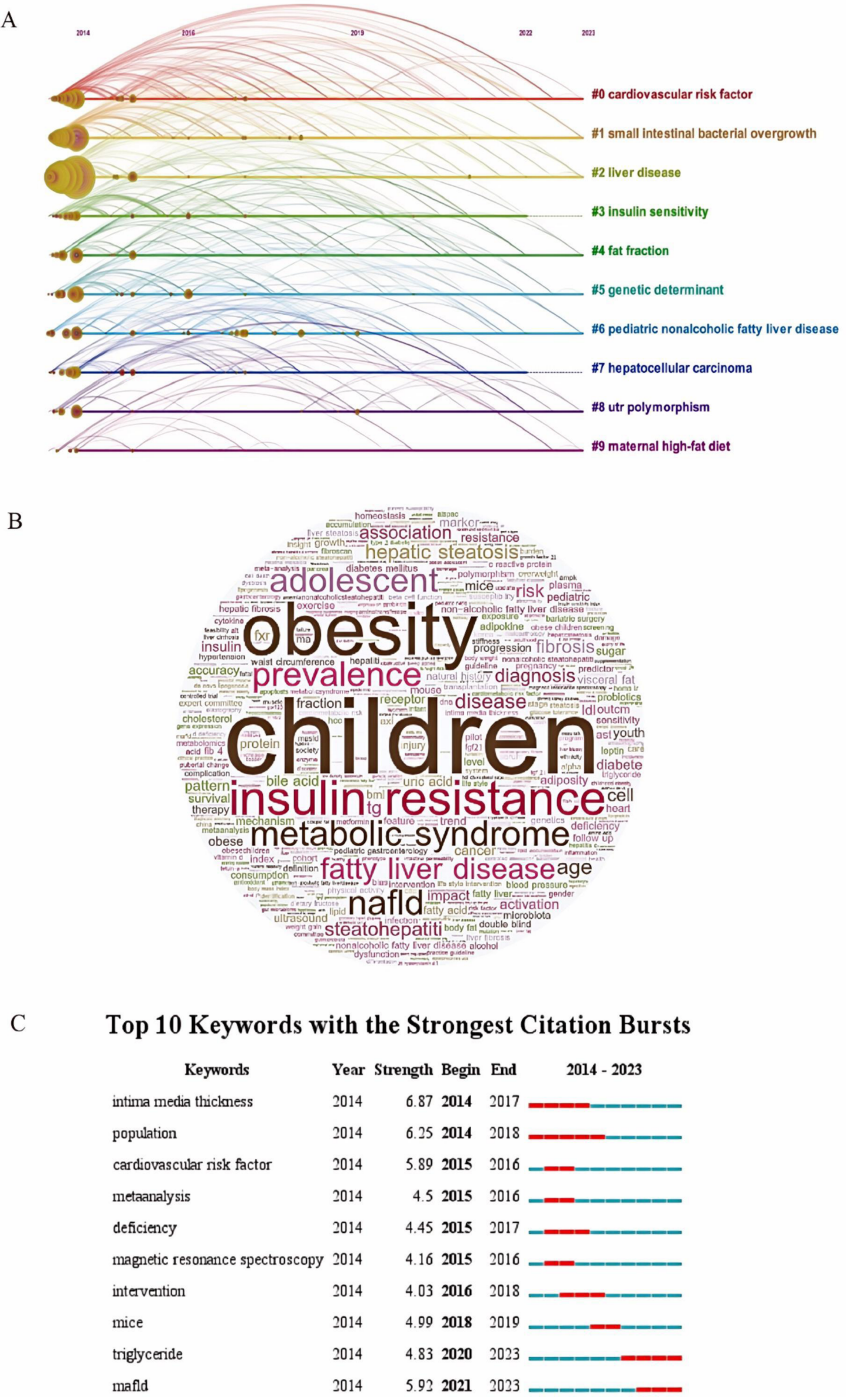
Analysis of cooccurring keywords in MAFLD publication references and burst detection from 2014-2023. (**A**) Displays a timeline of the most prominent research clusters, with cluster labels shown on the right side. (**B**) Presents a word cloud visualization of prominent terms in MAFLD-related publications. The relative size of each keyword corresponds to its frequency of occurrence in the analyzed literature. (**C**) The visualization highlights keywords exhibiting the strongest citation bursts, with red markers denoting periods of significantly increased usage frequency. Green represents a relatively unpopular period. The top 10 keywords are shown.

To clearly depict the shift in MAFLD research focus over the past 10 years, (Fig. **7A**) presents a temporal analysis of MAFLD research trends. The nodes in the timeline visualization represent the core publications within each research cluster, with the concentric citation tree rings proportionally indicating annual citation frequencies. It was found that the nonalcoholic steatohepatitis disease cluster starting in 2010 had the highest citation outbreak rate before 2016, followed by liver fibrosis. The research focus progressively transitioned toward MAFLD, as evidenced by recent publication trends. This shift is further corroborated by keyword frequency analysis (Fig. **7B**), where term prominence in pediatric MAFLD studies corresponds directly to occurrence frequency, with larger font sizes indicating higher prevalence in the literature. It was found that children, insulin resistance, and obesity were the keywords that appeared most frequently.

Keyword burst detection serves as an efficient bibliometric method for identifying emerging research hotspots and tracking conceptual evolution within a field. Fig. (**7C**) shows the top 10 keywords with the strongest eruptions in the study area from 2014-2023. The green line shows the 2014-2023 time frame, and the red line shows the time period during which the outbreak was sustained. Among the identified keyword bursts, those by the end of 2023 are led by intima-media thickness (intensity of 6.87), MAFLD (intensity of 5.92), and cardiovascular risk factors (intensity of 5.89).

## 4 DISCUSSION

### 4.1 Current Research Status

This bibliometric study analyzed 1,609 research articles on pediatric MAFLD, retrieved from the Web of Science Core Collection (2014-2023). Compared with previous broad-spectrum analyses, this targeted bibliometric study offers unique advantages by specifically focusing on the 2014-2023 period, which encompasses two pivotal transitions in pediatric fatty liver disease research: the implementation of the first NASPGHAN diagnostic guidelines and the subsequent shift from NAFLD to MAFLD nomenclature (2020 onward). This deliberate temporal framing enables us to capture the most recent research trends with higher temporal resolution and minimize historical heterogeneity in diagnostic criteria that affected earlier studies. With the help of VOSviewer and CiteSpace software, the study analyzes developmental trends in pediatric MAFLD from multiple perspectives, offering researchers a comprehensive assessment of recent hotspots and emerging directions.

Annual publications on pediatric MAFLD have shown a steady increase over the past decade, reflecting growing research interest in this field. As illustrated in Table **1**, the United States, Italy, and China are the three primary contributors to research on MAFLD in children. As demonstrated in Fig. (**3A**), the United States has a particularly strong record of collaboration with other countries, particularly China. These results demonstrate that the United States holds an important position and influence in the field of children's MAFLD. Concurrently, China is endeavoring to incorporate scientific innovation into the field of MAFLD. The Shanghai Birth Cohort Study database shows that MAFLD in children has become a public health concern in China. The analysis revealed that children with MAFLD had significantly greater BMI, body fat distribution indices (waist circumference, hip circumference, waist‒to‒height ratio, and waist-hip ratio), and liver stiffness measurements than those without MAFLD [32]. The prevalence of MAFLD in children has prompted China to allocate increased financial resources to research in this field. The enhancement of international collaboration is a salient trend, with the potential to expedite the generation of high-quality research outcomes. However, greater multinational and institutional cooperation remains necessary to advance this field.

The institutional collaboration network analysis indicates that the University of California San Diego (UCSD) occupies a central position in the research network, exhibiting both the highest publication productivity and the most extensive collaborative ties across all participating institutions. The author cooperation chart in Fig. (**3C**) shows that Valerio Nobili is the author with the most cooperation and communication. In the journal Nature Reviews Gastroenterology and Hepatology, Nobili advanced a unique understanding of the genetic factors, natural history, diagnosis, and therapeutic targets of the disease [33]. Nobili's study demonstrated that the primary burden of pediatric NAFLD lies in its progression to end-stage liver disease during adulthood. Specifically, non-alcoholic steatohepatitis can progress to advanced fibrosis and cirrhosis. The author highlighted the crucial importance of early diagnosis.

In the preceding decade, 9,463 investigators participated in research related to MAFLD. Among the cohort, 48 authors were identified as core contributors, each having authored ≥10 publications. Valerio Nobili from the Hepato- Gastroenterology Disease Unit at “Bambino Gesù” The Children's Hospital IRCCS, Rome, Italy, contributed the most articles (67), followed by Anna Alisi from the Research Unit of Molecular Genetics of Complex Phenotypes, also at the Bambino Gesù Children's Hospital IRCCS, Rome, Italy (57).

The Journal of Pediatric Gastroenterology and Nutrition has been cited most frequently (436 times) during the past decade, followed by the Journal of Pediatrics (270), Hepatology (245), and Gastroenterology (175). The Journal of Pediatrics (270), Hepatology (245), Gastroenterology (175), Plos One (155), Pediatric Obesity (148), Journal of Hepatology (138), World Journal of Gastroenterology (100), JAMA Pediatrics (81) and BMC Pediatrics (80) were also highly cited. Five of these journals are from the United States, while the others are from the Netherlands, China, Denmark, Australia, and England. Among the top 10 most-cited journals, JAMA Pediatrics had the highest average number of citations per paper (40.50), a figure potentially related to its high impact factor. The greater number of publications in PLoS One and Pediatric Obesity and their lower average citations per paper (3.52 and 3.15, respectively) indicate that papers published in these journals receive less attention and therefore fewer citations. The preponderance of journals from the United States, with six out of ten being from this country, suggests that the United States is a significant platform for the development of the field of children's MAFLD.

### 4.2 Research Hotspots

Compared to broader temporal analyses [29], our focused examination of the past decade (2014-2023) in pediatric MAFLD research more precisely captures critical features of the metabolic syndrome research paradigm shift - with cardiovascular metabolic risk factors (cluster #0) emerging as the most prominent research cluster. The strongest keyword burst for 'intima-media thickness' directly reflects the current research priority on early vascular pathology monitoring.

Keyword bursts are defined as keywords that are significantly cited in papers within a specified period. They are regarded as important indicators of research hotspots or emerging trends over time (Fig. **7A**) for the top 10 keywords with the strongest citations, which reveal potential hotspots in research on children's MAFLD over the past decade). Of particular note are the two keywords with the highest bursts, which began in 2014. The evolving research landscape has diminished the significance of some previously key terms in pediatric MAFLD studies.. Notably, the keyword 'intima-media thickness (IMT)' experienced a significant increase in citations in 2014 and currently holds the top position, with an intensity rating of 6.87. The extant literature pertaining to this keyword predominantly concentrates on the pathogenesis and prevention of MAFLD in children. Pacifico *et al.* [34] identified structural alterations in the vascular adventitia and perivascular adipose tissue (PAT) as potential contributors to early atherosclerotic development. Childhood obesity, a persistent public health concern, demonstrates significant tracking into adulthood and elevates long-term cardiovascular disease (CVD) risk [35] and is a significant contributing factor to the global burden of CVD. Endothelial dysfunction and adverse thickening of blood vessel walls are pivotal indicators of potential CVD risk factors [36-38].

A study on 109 obese children and 44 healthy children demonstrated that the severity of hepatic steatosis in obese children with liver degeneration was associated with increased right carotid intima-media thickness (cIMT) compared with healthy controls and obese children without liver degeneration [39]. A comparative study of 80 obese and 37 lean adolescents revealed that NAFLD-positive obese participants exhibited elevated left ventricular mass (LVM) and carotid intima-media thickness (cIMT), along with impaired insulin sensitivity. These findings suggest that obese adolescents with NAFLD and increased cIMT present an amplified cardiovascular risk profile characterized [40].

A prospective study comparing 89 pediatric patients meeting IDF criteria for metabolic syndrome (MetS) with 60 healthy controls revealed significantly higher mean carotid intima-media thickness (cIMT) and a greater prevalence of NAFLD in the MetS group. Compared with controls, subjects with MetS showed higher mean carotid intima-media thickness (cIMT) and greater NAFLD prevalence. The NAFLD prevalence correlated with both cIMT and IL-6 concentrations [41].

Cocited cluster analysis revealed that the terms most relevant to MAFLD studies in children by stratified cluster labels included #0 cardiovascular risk factor, #1 secondary intestinal bacterial overgrowth, #2 liver disease, #3 insulin sensitivity, #4 fat fraction, #5 genetic determinant, #6 pediatric nonalcoholic fatty liver disease, #7 hepatocellular carcinoma, #8 UTR polymorphism, and #9 maternal high-fat diet. It was found that there is a certain correlation between keyword bursts and cocited cluster analysis, in which there is a strong correlation between high-burst word IMT in keyword bursts and high-frequency label CVD in cocited cluster analysis. CVD constitutes a predominant determinant of both all-cause mortality and adverse clinical prognoses in patients with MAFLD [42, 43]. A national cohort study (N=8,962,813) with a median 10.1-year follow-up demonstrated that both NAFLD and MAFLD were independently associated with increased risk of incident CVD events [44]. In recent years, several meta-analyses [45-47] have examined the associations between NAFLD and cardiovascular events, and the results revealed that NAFLD increases the risk of CVD, including coronary heart disease (CHD), myocardial infarction (MI), atrial fibrillation (AF), congestive heart failure (CHF), and stroke. The study notably demonstrated that variations in diagnostic criteria did not significantly influence disease incidence rates [48, 49]. However, the analysis revealed that patients meeting exclusively MAFLD diagnostic criteria exhibited greater cardiovascular mortality risk compared to those fulfilling only NAFLD criteria [50]. It has not been determined whether this change in diagnostic criteria is strongly associated with cardiovascular events in children and young adults. Recent advances in innovative imaging modalities, particularly speckle tracking echocardiography (STE), hold significant potential for early identification of subclinical myocardial dysfunction in MAFLD patients across age groups [51].

### 4.3 Limitations

This study has several inherent limitations that warrant consideration. Primarily, the analysis was restricted to data extracted exclusively from the WoSCC SCI-E database, thereby excluding records from other platforms such as PubMed and Embase. This selective inclusion may result in incomplete representation of pediatric MAFLD/NAFLD publications over the past decade. Nevertheless, WoSCC remains the sole database providing comprehensive bibliometric metadata required for rigorous co-citation analysis using CiteSpace software.

Furthermore, the study was confined to English-language publications, potentially overlooking significant contributions from non-English-speaking regions. The exclusion of conference proceedings, preprints, and technical reports may additionally limit the generalizability of our findings, as emerging trends or applied research might be underrepresented. These methodological choices were nevertheless necessary to ensure data consistency and comparability, given that standardized citation metrics are more reliably available for peer-reviewed English articles. Future investigations can expand linguistic coverage and incorporate alternative sources (*e.g.* preprints) to obtain more comprehensive insights.

From a methodological perspective, while the co-citation clustering approach in CiteSpace is robust, it carries inherent limitations. Although algorithmic parameters (*e.g.*, time slicing, pruning, g-index) were standardized to enhance reproducibility, alternative clustering methodologies might yield slightly different structural representations. Subsequent research can benefit from cross-validating results using multiple bibliometric tools and expanded data sources to mitigate these potential biases.

Pediatric MAFLD bibliometric studies face insufficient child-specific data. Compared to adult research, pediatric studies are fewer, potentially reducing statistical power. Existing analyses often lack age stratification, masking developmental-stage differences in pathology and risk factors [52, 53]. Future work should prioritize age-specific analyses to better characterize pediatric MAFLD heterogeneity.

## CONCLUSION

This study conducted a systematic bibliometric and visual analysis of the scientific literature pertaining to MAFLD in pediatric populations over the past decade. Through the application of these analytical methods, researchers can efficiently identify emerging research trends and delineate key knowledge domains within the rapidly expanding body of MAFLD-related publications. The findings provide significant insights that may inform the establishment of future research priorities and the optimization of investigative strategies in the field of pediatric MAFLD. Furthermore, this analysis offers valuable information that could facilitate the identification of potential collaborative opportunities with relevant research institutions and domain experts, thereby fostering interdisciplinary cooperation in this important area of pediatric hepatology research.

## Figures and Tables

**Fig. (1):**
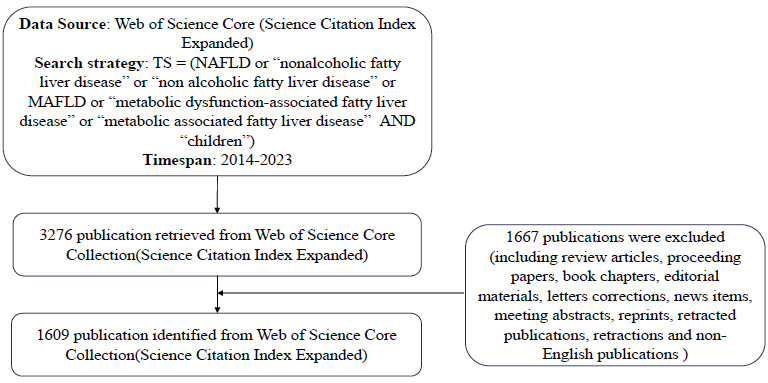
Flowchart of included and excluded publications.

**Fig. (2):**
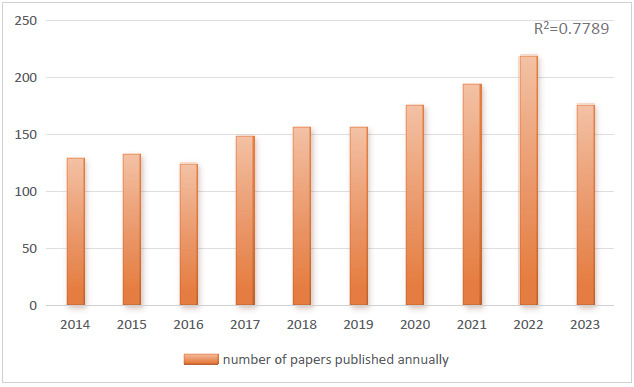
Analysis of the number and trends of published papers on MAFLD research on children, 2014-2023. The X-axis indicates the year of publication, and the Y-axis indicates the number of articles on children's MAFLD published in that year.

**Fig. (5):**
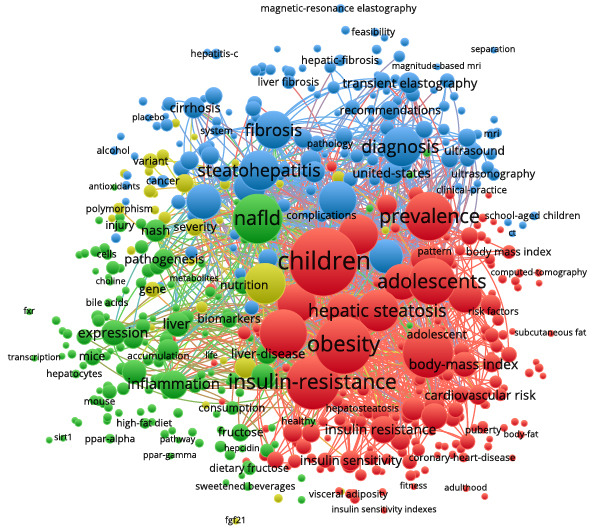
Visualization of keyword co-occurrence analysis (total link strength values = 59334).

**Fig. (6):**
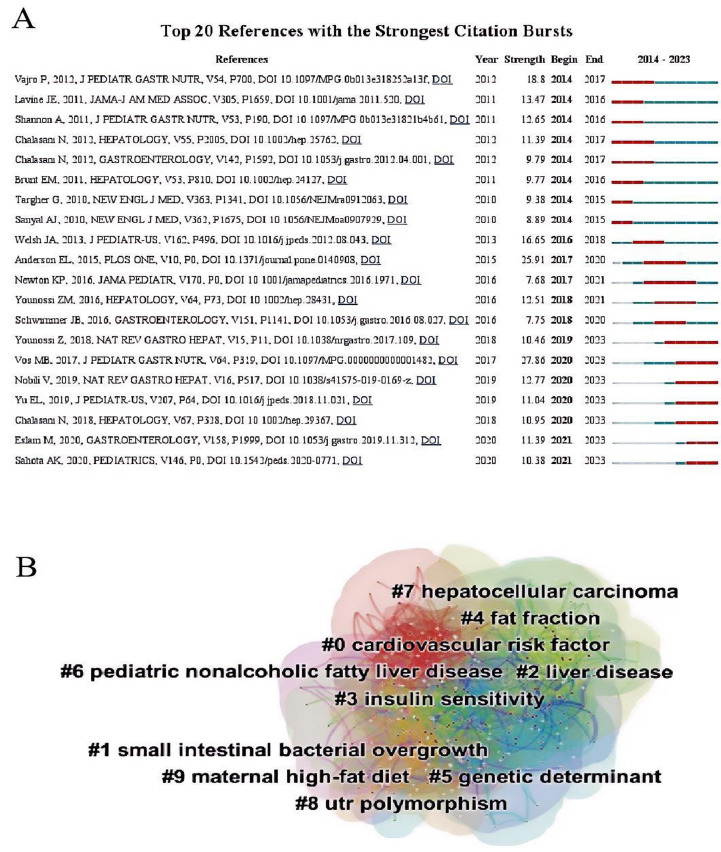
Reference cocitation network analysis of publications on MAFLD research from 2014-2023. (**A**) References with the strongest burst strength of the citing articles on MAFLD and Children research between 2014 and 2023. Red segments denote periods of significant citation increase. Green segments represent baseline citation frequency. The top 20 references with the strongest citation bursts are shown. (**B**) Visualizing the CiteSpace generated co-citation cluster network shows the 9 most prominent topic clusters in the children’s MAFLD study.

**Table 1:** The top 10 productive countries and institutions for MAFLD research on children from 2014-2023.

Rank	Country	Count	Rank	Institution	Count
1	USA	523	1	Univ Calif San Diego	71
2	ITALY	198	2	Emory Univ	48
3	PEOPLES R CHINA	188	3	Bambino Gesu Pediat Hosp	47
4	GERMANY	88	4	Cincinnati Childrens Hosp Med Ctr	47
5	ENGLAND	82	5	Univ Cincinnati	46
6	CANADA	69	6	Columbia Univ	44
7	SPAIN	67	7	Washington Univ	34
8	SOUTH KOREA	63	8	Univ Roma La Sapienza	31
9	TURKEY	49	9	Univ Colorado	31
10	NETHERLANDS	43	10	Karolinska Inst	26

**Table 2:** The top 10 most active journals that published articles on MAFLD research from 2012-2021 (sorted by total citations).

Rank	Journal Title	Frequency	Total citations	Average citation per paper	Impact factor (2023)	Country	JCR Quartile
1	Journal of Pediatric Gastroenterology and Nutrition	63	436	6.92	2.4	USA	Q1 (Pediatrics); Q2(Gastroenterology)
2	Journal of Pediatrics	34	270	7.94	3.9	USA	Q1(Pediatrics)
3	Hepatology	36	245	6.81	13.0	USA	Q1(Gastroenterology & Hepatology)
4	Gastroenterology	15	175	11.67	26.3	USA	Q1(Gastroenterology & Hepatology)
5	PLoS ONE	44	155	3.52	2.9	USA	Q2(Multidisciplinary Sciences)
6	Pediatric Obesity	47	148	3.15	2.7	England	Q1(Nutrition & Dietetics) Q2(Pediatrics)
7	Journal of Hepatology	22	138	6.27	26.8	Netherlands	Q1(Gastroenterology & Hepatology)
8	World Journal of Gastroenterology	24	100	4.17	4.3	China	Q2(Gastroenterology & Hepatology)
9	JAMA Pediatrics	2	81	40.50	24.7	USA	Q1(Pediatrics)
10	BMC Pediatric	17	80	4.71	2.0	England	Q2(Pediatrics)

## LIST OF ABBREVIATIONS

MAFLD: Metabolic Dysfunction-related Fatty Liver Disease
UCSD: University California San Diego
NAFLD: Non-alcoholic Fatty Liver Disease
T2DM: Type 2 Diabetes Mellitus
GERD: Gastroesophageal Reflux Disease
OSA: Obstructive Sleep Apnea
WHO: World Health Organization
BMI: Body Mass Index
HCC: Hepatocellular Carcinoma
SCI-E: Science Citation Index-Expanded
WoSCC: Web of Science Core Collection
PAT: Perivascular Adipose Tissue
CVD: Cardiovascular Disease
IMT: Intima-Media Thickness
LVM: Left Ventricular Mass
MetS: Metabolic Syndrome
cIMT: Carotid Intima-Media Thickness
CHD: Coronary Heart Disease
MI: Myocardial Infarction
AF: Atrial Fibrillation
CHF: Congestive Heart Failure
STE: Speckle Tracking Echocardiography
